# Hépatite virale B: aspects cliniques, paracliniques et évolutifs dans le service d’Hépato Gastroentérologie de l’Hôpital Aristide Le Dantec: à propos de 728 cas

**DOI:** 10.11604/pamj.2018.31.82.14725

**Published:** 2018-10-03

**Authors:** Salamata Diallo, Marie Louise Bassène, Mamadou Ngoné Gueye, Mame Aissé Thioubou, Daouda Dia, Mouhamadou Mbengue, Mamadou Lamine Diouf

**Affiliations:** 1 Service d’Hépato-gastro-entérologie, Hôpital Aristide Le Dantec, Dakar, Sénégal; 2 Service d’Hépato-gastro-entérologie, Hôpital Général de Grand Yoff, Dakar, Sénégal

**Keywords:** Hépatite B, Afrique sub saharienne, Tenofovir, Hepatitis B, sub-Saharan Africa, Tenofovir

## Abstract

L’hépatite virale B constitue un problème majeur de santé publique en Afrique subsaharienne avec environ 65 millions de porteurs chroniques et 56.000 décès par an. Notre étude avait pour but d’étudier les aspects épidémiologiques, paracliniques, thérapeutiques, évolutifs des patients atteints d’hépatite virale B suivis dans notre service et de décrire leurs profils sérologiques. Nous avons réalisé une étude rétrospective, longitudinale de 2010 à 2014 dans le service d’hépato gastroentérologie de l’hôpital Aristide Le Dantec de Dakar. Nous avons inclus tous les patients suivis en ambulatoire ou hospitalisés qui avaient une positivité de l’AgHBs. Nous avons colligé 728 cas de patients infectés par le virus de l’hépatite B. Ils étaient constitués de 7 cas d’hépatite aiguë, de 442 cas d’infections chroniques, de 161 cirrhotiques et de 118 cas de carcinome hépatocellulaire. L’âge moyen était de 33 ans [14 - 83 ans] avec un sex-ratio de 2,2. Les circonstances de découverte étaient représentées par le dépistage systématique (26,2%), les douleurs de l’hypochondre droit (23%) et le don de sang (18,6%). Il y avait 59 patients monoinfectés par le virus de l’hépatite B qui étaient au stade d’hépatite chronique active. Les porteurs inactifs étaient au nombre de 118. Par insuffisance d’exploration liée à des contraintes économiques, le statut sérologique était indéterminé chez 252 patients. Un traitement antiviral B n’a pu être institué que chez 58 patients. Sous Ténofovir, la réponse virologique et biochimique était respectivement de 85% et 100% après 120 semaines de traitement. Le virus de l’hépatite B est une cause majeure d’hépatopathie au Sénégal.

## Abstract (en)

Viral Hepatitis B is a major public health problem in sub-Saharan Africa accounting for approximately 65 million of chronic carriers and 56.000 deaths per year. Our study aims to investigate the epidemiological paraclinical, therapeutic and evolutionary features of viral hepatitis B in patients followed up in our Department and to describe their serological profiles. We conducted a retrospective, longitudinal study in the Hepatogastroenterology Department at the Aristide Le Dantec Hospital in Dakar from 2010 to 2014. We included all HBsAg positive patients followed up on an ambulatory basis or hospitalized. We collected data from 728 medical records of patients infected with Hepatitis B virus: 7 cases of acute hepatitis, 442 cases of chronic infections, 161 cases of cirrhosis and 118 cases of hepatocellular carcinoma. The average age of patients was 33 years [14 - 83 years] with a sex ratio of 2.2. The circumstances in which it was diagnosed included systematic screening (26.2%), right hypochondrium pain (23%) and donation of blood (18.6%). Fifty nine were Hepatitis B virus mono-infected and had chronic active hepatitis. Inactive carriers were 118. Serological status was undetermined in 252 patients due to clinical examination inadequacy related to economic constraints. Antiviral Hepatitis B treatment wasn’t performed in 58 patients. Patients’ virologic and biochemical response after 120 weeks of treatment with Tenofovir was 85% and 100% respectively. Hepatitis B virus is a major cause of liver disease in Senegal.

## Introduction

L’hépatite virale B constitue un problème mondial de santé publique [1]. L’Afrique subsaharienne est située dans une zone de forte endémicité avec 65 millions de porteurs chroniques et 56.000 décès par an [2, 3]. L'histoire naturelle de l'infection par le virus de l’hépatite B est caractérisée par la diversité des profils cliniques avec les hépatites aigues, les hépatites chroniques et les complications que sont la cirrhose ou le carcinome hépatocellulaire (CHC). La prise en charge de cette infection dans nos régions est confrontée à de nombreuses difficultés d’ordre socio-économiques et culturelles. Les objectifs de notre travail étaient de décrire les aspects épidémiologiques, cliniques, paracliniques, thérapeutiques et évolutifs des patients atteints d’hépatite virale B suivis dans notre service et de décrire leurs profils sérologiques.

## Méthodes

Nous avons réalisé une étude rétrospective, longitudinale sur une période de 60 mois (du 01 janvier 2010 au 31 décembre 2014) dans le service d’hépatogastroentérologie de l’hôpital Aristide Le Dantec de Dakar. Nous avons inclus tous les patients suivis en ambulatoire ou hospitalisés qui avaient une positivité de l’AgHBs. La collecte des données s’est faite par consultation des dossiers médicaux des patients. Une fiche d’enquête a été établie pour chaque patient et comportait les paramètres suivants: 1) **Les caractéristiques sociodémographiques:**âge, sexe, profession, adresse, statut matrimonial; 2) Les caractéristiques cliniques, les données biologiques, virologiques, histologiques, échographiques et scannographiques; 3) **Les schémas thérapeutiques:**molécules, observance, durée, effets secondaires; 4) Les aspects évolutifs cliniques et paracliniques. La saisie et l’analyse des données étaient faites avec le logiciel Epi-data version 3.1. Les moyennes et les pourcentages ont été comparés à l’aide du test du chi2, du test exact de Fisher suivant leurs conditions d’applicabilité. Toute différence inférieure à 0,05 était considérée comme statistiquement significative.

## Résultats

Durant la période d’étude, nous avons colligé 728 cas de patients infectés par le virus de l’hépatite B. Ils se répartissaient en 7 cas d’hépatite aiguë, 442 cas d’infection chronique, 161 cirrhotiques et 118 cas de carcinome hépatocellulaire (CHC). Il avait 502 hommes et 226 femmes soit un sex-ratio de 2,2. L’âge moyen était de 33 ans [14 et 83 ans]. Vingt-sept patients avaient des antécédents familiaux de CHC (3,7%). Les principales circonstances de découverte étaient représentées par le dépistage systématique (26,2%), les douleurs abdominales (23%), le don de sang (18,6%) (Tableau 1). L’examen physique était normal chez 429 patients (58,9%) et objectivait une ascite dans 18,7% des cas (Tableau 1). Il y avait 59 cas d’hépatite chronique active au virus de l’hépatite B (VHB) seul et 118 cas de portage inactif. Par insuffisance d’exploration liée à des difficultés socio-économiques, le statut sérologique était indéterminé chez 252 patients (34,6%). Les anticorps antivirus de l’hépatite D (VHD) étaient présents chez 21 patients (6,3 %) et la charge virale D était positive chez 5 patients (1,5%). Les anticorps antivirus de l’hépatite C (VHC) étaient positifs chez 12 patients (3,3%) et la virémie était détectable chez 3 patients (0,8 %). La sérologie rétrovirale était positive chez 2 patients (0,6%).

**Tableau 1:** Caractéristiques sociodémographiques et cliniques des patients

**Circonstances de découverte**		
Ictère	116	16 %
Douleurs abdominales	170	23 %
Ascite	60	8,2 %
Hémorragie digestive	70	9,6 %
Don de sang	135	18,6 %
Dépistage systématique	191	26,2 %
Grossesse	39	5,3%
**Manifestations cliniques**		
Asthénie	184	25 %
Amaigrissement	199	27,3 %
Encéphalopathie hépatique	15	2 %
Hépatomégalie	130	17,8 %
Atrophie hépatique	30	4,1 %
Splénomégalie	99	13,5 %
CVC	32	4,4 %
OMI	25	3,4 %
Ascite	136	18,7 %

CVC: circulation veineuse collatérale, OMI: œdèmes des membres inférieurs

Chez les patients atteints d’hépatite chronique active, le taux moyen d’alanine aminotransférase (ALAT) et la charge virale moyenne étaient respectivement de 4,3 N et 4,5 log10UI/ml (Tableau 2). Une ponction biopsie hépatique était réalisée chez 32 patients et objectivait une fibrose modérée à sévère dans 44% des cas (≥ A2F2). Une évaluation non invasive de la fibrose par l’association Fibrotest-Actitest était faite chez 18 patients et montrait une fibrose modérée à sévère dans 55,5%. L’échographie abdominale était normale chez 311 patients et montrait un foie hétéro-multinodulaire dysmorphique dans 31%, des signes d’hypertension portale dans 15,6% et un nodule tumoral dans 7,2%. Le taux moyen d’alfafoetoproteine (AFP) était de 3655 UI/ml [1-642430 UI/ml] chez les patients atteints d’hépatocarcinome. Cinquante-deux pour cent des patients cirrhotiques étaient classés au stade B de CHILD-PUGH et 26% au stade C de CHILD-PUGH.

**Tableau 2:** Caractéristiques biologiques et virologiques des patients

	Hépatite aigue	Porteur inactif	Hépatite chronique active	Cirrhose	CHC
ASAT	19,7 N	0,6 N	3,2 N	3,5 N	4,6 N
ALAT	24,7 N	0,5 N	4,3 N	1,5 N	2,3 N
TP	59%	87 %	88%	60%	64%
Albumine g/l	-	41,2	41,7	29,9	31,8
Bilirubine	4,5 N	0,7 N	1,8 N	2,3 N	4,4 N
charge viral B Log10 UI/ ml	-	2,6	4,5	3,6	3,8
AgHBe- /Ac anti HBe + Nombre de patients	-	-	54	38	8
Perdus de vue Effectif	4	63	26	116	55
Décès	0	0	0	8	63

N: normal, ASAT: aspartate aminotransférase, ALAT: alanine aminotransférase, TP: taux de prothrombine

Un traitement antiviral B n’a pu être institué que chez 58 patients. Ce traitement était à base de Ténofovir chez 55 patients (18 patients présentant une hépatite chronique active et 37 cirrhotiques). La posologie était de 300 mg par jour en prise unique per os avec une durée moyenne de traitement de 22 mois [4-50 mois]. Un traitement par interféron pégylé était administré chez 3 patients. Il s’agissait d’un patient de 17 ans mono-infecté par le VHB et de 2 patients présentant une co-infection BD dont 1 au stade de cirrhose compensée. La posologie était de 180 µg par semaine par voie sous cutanée. La durée du traitement était de 12 mois chez le patient monoinfecté par le VHB. Il était respectivement de 4 et 12 mois chez les patients co infectés B-D. Les effets secondaires de l’interféron ont entrainé l’arrêt du traitement au bout de 4 mois chez un des patients co-infecté B-D. Un traitement antiviral n’a pas été institué chez 5 patients co-infectés VHB-VIH ou VHB-VHC car perdus de vue. Un traitement symptomatique à base de bêta bloquant, d’antibiotiques, de diurétiques et d’antalgiques a été administré chez les cirrhotiques et les patients atteints d’hépatocarcinome. Une ligature des varices œsophagiennes a été réalisée chez 50 patients et une transfusion sanguine iso groupe et iso rhésus chez 19 patients.

Un patient présentant un CHC de 7 cm du lobe gauche a bénéficié d’une hépatectomie segmentaire. Au cours du traitement antiviral par Ténofovir, l’évolution clinique était favorable avec une régression des manifestations cliniques chez les cirrhotiques. Une surveillance régulière de la charge virale a pu être faite chez 40 patients. Elle était indétectable chez 34 patients après 120 semaines de traitement (Tableau 3). Six patients ont eu une baisse significative de la charge virale cependant elle restait détectable après 120 semaines. Tous les patients ont eu une normalisation des transaminases sous Ténofovir (Tableau 3). Le taux de créatinine et de phosphore était normal au cours du traitement par Ténofovir. La surveillance échographique couplée au dosage de l’AFP chez les patients cirrhotiques a permis de détecter un nodule de CHC de 2 cm chez un patient sous Ténofovir. Ce CHC a été traité par alcoolisation avec bonne évolution clinique. Trois cas de séroconversion HBs ont été retrouvés chez les patients suivis pour hépatite aigue. En l’absence de traitement antiviral, il y avait 69 cas de décès. Les circonstances de décès étaient représentées par l’infection du liquide (19%), l’encéphalopathie hépatique (45%), l’ascite réfractaire (12%), les hémorragies digestives (24%) (Tableau 2).

**Tableau 3:** Traitement antiviral

		Ténofovir	Interféron pégylé
Nombre de patients		55	3
Obervance	Bonne	48	3
	Mauvaise	7	0
Effets secondaires		Asthénie Douleurs abdominales	Syndrome pseudo grippal Douleurs abdominales Rash cutané thrombopénie
Réponse virologique		85 %	66 %
Réponse biochimique		100%	66 %
Séroconversion HBe		0	2
Séroconversion HBs		0	1

## Discussion

Dans notre étude, la population était constituée principalement d’adultes jeunes avec un âge moyen de 33 ans (extrêmes 14-83 ans). Ces résultats sont conformes avec les données de la littérature. Le Sénégal est un pays de forte endémicité du VHB avec un taux de portage chronique estimé à 11% [4]. Dans notre série, 27 patients avaient des antécédents familiaux de CHC (3,7%). Les antécédents familiaux de CHC constituent un des facteurs de risque du carcinome hépatocellulaire. Le suivi et l’indication thérapeutique doivent les prendre en considération. Le dépistage du VHB dans la fratrie et dans la descendance d’un sujet atteint d’hépatite virale B est important et doit être systématique. Le portage inactif du VHB témoin du contrôle de la maladie par la réponse immune était retrouvé chez 118 patients (16,2%) Une étude réalisée au Burkina Faso trouvait 23,3% de porteurs inactifs [5].

Le pronostic est habituellement favorable chez ces patients, cependant il existe un risque très faible de développer une cirrhose ou un CHC. Le traitement n’est pas recommandé mais le suivi reste obligatoire [6]. Une hépatite chronique active était présente chez 8,1% des patients. Chez ces patients, il y avait une cytolyse moyenne avec un taux moyen de transaminase à 4,3 N. Une étude réalisée en Côte d’Ivoire trouvait des valeurs plus basses avec un taux de transaminases de 1,9 N [7]. Des taux relativement peu élevés d’ADN viral B ont été retrouvés chez la majorité de nos patients avec une moyenne de 4,5 log10UI/ml. La sévérité des hépatopathies virales B et la survenue de la cirrhose et de ses complications sont précipitées par la persistance d’une multiplication virale, génératrice de l’activité nécrotico-inflammatoire et responsable de la morbi-mortalité [8]. L’instauration d’un traitement antiviral est souvent indiquée à ce stade de l’hépatite chronique B. Une proportion importante de patients avait un statut sérologique indéterminé (32%). Ce pourcentage important pourrait être expliqué par: le coût élevé des explorations complémentaires (charge virale, bilan de co-infection virale). La prise en charge de l’hépatite virale B à grande échelle dans nos pays passe par la subvention ou la diminution du coût des explorations complémentaires et la mise en place de mesures sociales d’accompagnement. Dans notre étude, il y avait 161 cirrhotiques et 118 CHC. Dans les pays en développement, l’hépatite B est souvent découverte au stade de complications. Les traitements par inhibiteurs nucléosidiques et nucléotidiques permettent une stabilisation voire une régression des lésions de fibrose et une diminution des complications [8]. Cinquante-cinq patients ont bénéficié d’un traitement antiviral à base de Ténofovir conformément aux recommandations internationales [9].

Dans notre série, la tolérance rénale était bonne. Le bilan phosphocalcique était normal. Le Ténofovir a un bon profil sécuritaire. La réponse virologique était de 85% après 120 semaines de traitement. Marcellin trouvait dans leur étude 74% de réponse virologique après 144 semaines de traitement par Ténofovir chez des cirrhotiques [10]. La réponse biochimique était de 100% après 120 semaines de traitement. L’évolution sous Ténofovir était marquée par une amélioration sur le plan clinique dans tous les cas. Les études ont montré que la suppression virale continue est corrélée à l'amélioration de la réponse clinique. Aucun cas de séroconversion anti-HBe ou de perte de l’AgHBs n’a été noté au cours du traitement. L’apparition d’un cas de CHC chez un cirrhotique sous Ténofovir souligne l’importance de la surveillance échographique chez ces patients. Il est établi que le traitement antiviral diminue le risque de CHC sans l’annuler [8]. La limite actuelle du traitement de l’hépatite B par les analogues nucléosidiques et nucléotidiques réside dans la durée souvent indéterminée du traitement. Des molécules ciblant l’ADN super enroulée ou le système immunitaire sont en cours d’étude.

## Conclusion

Le virus de l’hépatite B est une cause majeure d’hépatopathie au Sénégal. Le traitement antiviral B apporte un bénéfice clinique net avec une baisse des complications liées à l’infection virale B. La vaccination universelle couplée au traitement antiviral permettra dans l’avenir la maitrise de la morbidité et de la mortalité liée au virus de l’hépatite B.

### Etat des connaissances actuelles sur le sujet

- L’hépatite virale B constitue un problème de santé publique en Afrique sub saharienne et elle est responsable d’une importante morbi-mortalité par le biais de ses complications;
- La prise en charge de cette affection dans nos régions est confrontée à de nombreuses difficultés socio-économiques et culturelles.

### Contribution de notre étude à la connaissance

- L’histoire naturelle de l’hépatite chronique virale B est caractérisée par la diversité des profils sérologiques et la connaissance de ces profils joue un rôle fondamental dans la prise en charge des patients notamment pour la décision d’instauration du traitement antiviral; notre étude a permis de préciser ces différents profils chez les patients;
- Notre étude permet de mettre en exergue l’efficacité du traitement antiviral par Ténofovir dans nos régions.

## Conflits d’intérêts

Les auteurs ne declarent aucun d’intérêts.
